# Clinical features, MRI, and 18F‐FDG‐PET in differential diagnosis of Parkinson disease from multiple system atrophy

**DOI:** 10.1002/brb3.1827

**Published:** 2020-09-17

**Authors:** Ping Zhao, Benshu Zhang, Shuo Gao, Xin Li

**Affiliations:** ^1^ Department of Neurology Second Hospital of Tianjin Medical University Tianjin China; ^2^ Department of Neurology General Hospital of Tianjin Medical University Tianjin China; ^3^ Department of Nuclear Medicine General Hospital of Tianjin Medical University Tianjin China

**Keywords:** ^18^F‐FDG‐PET, differential diagnosis, MRI, multiple system atrophy, neurodegenerative disease, Parkinson's disease

## Abstract

**Objective:**

This study aimed to differentiate the variations in the clinical characteristics, MRI irregularity, and glucose metabolism on ^18^F‐FDG‐PET for the differential diagnosis of Parkinson's Disease (PD), MSA with predominant Parkinsonism (MSA‐P), and MSA with predominant cerebellar features (MSA‐C).

**Methods:**

Thirty PD patients, 22 MSA‐P patients, and 28 MSA‐C patients received an MRI and 20 PD patients, 11 MSA‐P patients, and 13 MSA‐C patients received ^18^F‐FDG‐PET.

**Results:**

Firstly, we found that the clinical data presented a tremor at rest, bradykinesia, and postural instability that was predominated in PD (100%), MSA‐P (86.4%), and MSA‐C (53.6%) patients, respectively. Then, we used MRI analyses and found that putamina atrophy and hyperintensive rim (T_2_WI) were characteristic features in MSA‐P and cerebellar atrophy, the “hot cross bun” sign and signal rise in the middle cerebellar peduncle were more obvious in MSA‐C. To further explore the distinctions among the 3 diseases, we also used 18F‐FDG‐PET technology for our examination and found a decrease in glucose metabolism in the parietal area for Parkinson's Disease (PD), in the bilateral putamen for MSA‐P, and in the bilateral cerebellum for MSA‐C.

**Conclusion:**

This study identified the distinctive features of the clinic symptoms, MRI irregularity, and glucose metabolism on ^18^F‐FDG‐PET, which provided a new basis for the differential diagnosis of Parkinson's Disease (PD), MSA with predominant Parkinsonism (MSA‐P), and MSA with predominant cerebellar features (MSA‐C).

## Graphical abstract

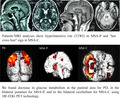

## 1 INTRODUCTION

Parkinson's disease (PD) is a progressive neurodegenerative disease characterized by certain clinical features, such as bradykinesia, rigidity, and resting tremor, and is associated with progressive neuronal loss in substantia nigra and other brain regions. The diagnosis of PD can be straightforward in patients with typical clinical presentation of cardinal signs, but misdiagnosis of PD is not uncommon. In a clinical‐pathological study, the accuracy of a clinical diagnosis of PD is not high, with only 76% of postmortem confirmed cases being diagnosed correctly (Rajput, Rozdilsky, & Rajput, 1991). The most common misdiagnoses are related to Atypical Parkinson's disease (APD) such as multiple system atrophy, progressive supranuclear palsy, and corticobasal degeneration (Horvath, Burkhard, Bouras, & Kövari 2013; Levin, Kurz, Arzberger, Giese, & Höglinger 2016). Since prognosis and treatment options of PD and APD are substantially different, differential diagnosis of PD from APD is critical for clinicians.

Multiple system atrophy (MSA), one of the most common APD, is an adult‐onset neurodegenerative disease characterized by a combination of parkinsonism, autonomic disorder, and cerebellar ataxia (Coon et al., 2015). Based on the predominant symptoms of the disease, MSA is categorized into two forms of MSA: MSA with predominant Parkinsonism (MSA‐P) and MSA with predominant cerebellar features (MSA‐C) (Gilman et al., 2008). The differential diagnosis of MSA from PD can be challenging since MSA shares some clinical features with PD, especially at the early stage of the disease (McKay & Cheshire, 2018). In addition, some features, which can differentiate MSA from PD, such as poor responsiveness to levodopa, autonomic dysfunction, and cerebellar incoordination, may take several years to occur (Bhatia, Stamelou, Fanciulli, & Wenning, 2015). Therefore, it is difficult to differentiate these disease using clinical criteria alone, and efforts have been made to improve the accuracy of the differential diagnosis with imaging methods.

Many imaging methods such as magnetic resonance imaging (MRI) and positron emission tomography (PET) have been used to differentiate PD from MSA and other atypical Parkinsonism (Kwon, Choi, Kim, Lee, & Chung, 2007, 2008; Meyer, Frings, Rücker, & Hellwig 2017; Walker et al., 2017). The differential diagnosis can be assisted by the characteristic features of MSA revealed by MRI, such as atrophy of the brainstem and cerebellum, and putaminal hyperintensive rim and “hot cross bun” sign (Hughes, Daniel, Ben‐Shlomo, & Lees, 2002; Poewe & Wenning, 2002). However, it remains unclear whether these MRI findings are valid for differentiation of MSA from PD. In addition, the MRI findings for differentiation between MSA‐P and MSA‐C have not well studied.

Multi‐tracer PET including ^18^F‐dopamine PET, dopamine transporter PET, dopamine receptor PET, and tau PET increasingly used for diagnosis and differentiation of PD and APD. However, an overlap between PD and MSA in these PET images limits their usefulness in the differential diagnosis (Koga, Ono, Sahara, Higuchi, & Dickson, 2017; Niccolini & Politis, 2016). Metabolic brain imaging by ^18^F‐fluorodeoxyglucose (^18^F‐FDG) PET has showed characteristic reduction in glucose metabolism in the lentiform nucleus and cerebellum in MSA patients (Eckert & Eidelberg, 2004; Meyer et al., 2017), which can differentiate MSA patients from PD patients, especially at the early disease stages when no characteristic features occur on MRI. However, the patterns of glucose metabolism on ^18^F‐FDG‐PET in PD, MSA‐P, and MSA‐C patients have not been well established yet.

In this study, we aimed to characterize the differences in clinical features, MRI abnormality, and glucose metabolism on ^18^F‐FDG‐PET for differential diagnosis among PD, MSA‐P, and MSA‐C.

## 2 SUBJECTS AND METHODS

### 2.1 Subjects

This study included 30 PD patients (16 males and 14 females), 22 MSA‐P patients and 28 MSA‐C patients, and 44 age‐matched healthy controls. All these patients underwent head MRI, among them 20 PD patients (14 males, age 62.5 ± 11.7 years), 11 MSA‐P patients (5 males, age 67.4 ± 6.1 years), and 13 MSA‐C patients (6 males, age 59.8 ± 8.7 years) underwent both MRI and ^18^F‐FDG‐PET examinations. The study was approved by the Medical Ethics Committee of the Tianjin Medical University, and all subjects gave their informed consent. The PD patients were diagnosed according to MDS clinical diagnostic criteria for Parkinson's disease (Postuma et al., 2015), and MSA patients were diagnosed according to the Second consensus statement on the diagnosis of multiple system atrophy by Gilman et al. (2008). The clinical data were listed in Table 1. Patients with drug‐induced Parkinsonism, vascular Parkinsonism, supranuclear palsy, corticobasal degeneration, and dementia with Lewy bodies were excluded. Patients were evaluated by the unified Parkinson's disease rating scale (UPDRS) part I, II and III, and Hoehn‐Yahr stage.

**TABLE 1:** Clinical characteristics of PD patients, MSA‐P patients, MSA‐C patients

Groups	PD (*n* = 30)	MSA‐P (*n* = 22)	MSA‐C (*n* = 28)
Age (years)	62.47 ± 7.94	59.32 ± 9.71	58.82 ± 7.19
Male (%)	16 (53.33)	14 (63.64)	12 (42.86)
Disease duration (months)	42.93 ± 22.11	35.55 ± 18.93	35.82 ± 21.59
UPDRS motor score	17.77 ± 6.98	26.91 ± 8.60*	24.68 ± 6.06*
H‐Y stage	1.93 ± 0.72	2.73 ± 0.63*	2.76 ± 0.60*

* *p* < .05 versus PD.

### 2.2 Magnetic resonance imaging

The magnetic resonance imaging (MRI) was performed in all patients using MRI device (GE, USA), and a circular polarized head coil. All patients had axial T1‐weighted Fluid‐Attenuated Inversion Recovery Sequence (T1FLAIR), T2‐weighted images (T2WI), sagittal T1FLAIR, and coronal T2WI. T1FLAIR was performed as follows: repetition time (TR), 2000–2100 ms; inversion time(TI), 750 ms; and echo time (TE), 10–30 ms. T2WI was performed as follows: TR. 4000–5000 ms; TE, 100–160 ms. The slice thickness was set at 6 mm with a interslice gap of 2 mm.

MRI images were obtained at pons, cerebellum, basal ganglia and cerebrum, and were evaluated by two blinded radiologists, focusing on the changes in infratentorial parameters (cerebellar atrophy, signal increase in the middle cerebellar peduncle, and “hot cross bun” sign), and supratentorial parameters (signal decrease in the basal ganglia, putaminal hyperintensive rim, and cortical atrophy).

### 2.3 ^18^F‐FDG‐PET imaging

The PET scans were performed in a 3‐dimensional mode using GE Discovery LS PET/CT system. A computerized tomography image was obtained for attenuation correction. The subjects fasted for at least 6 hr and had no psychiatric drugs for at least 2 weeks before the PET scanning procedure. Subjects lied supine in a dark and quiet room for 15 min and were administered with an intravenous bolus injection of 185–370 MBq (5–10 mCi/) ^18^F‐FDG. After 40 min of rest, the heads of subjects were held with fixation strips, and ^18^F‐FDG scan was obtained. The scanner was aligned parallel to the orbitomeatal line using a laser beam. Series of PET images were acquired using a rotating position emission detector. Three‐dimensional data acquisition mode was performed, and cross‐sectional, coronal, and sagittal images were collected with slice thickness of 2 mm collected in 128 × 128 matrix.

The ^18^F‐FDG‐PET results were evaluated by two nuclear medicine physicians blinded to the clinical diagnosis of the patients. Changes of ^18^F‐FDG metabolism in regions of interest, including cerebrum, cerebellum, caudate nucleus, lenticular nucleus, and thalamus, were analyzed using visual inspection and SPM methods. For SPM methods, the data were converted from DICOM file into Analyze 7 format. Then, the images were spatially transformed and analyzed with SPM2 running on MATLAB.

### 2.4 Statistical analysis

Analyses were performed using SPSS 22. All values were presented as mean and standard deviation. Student *t* test was used to compare the difference between PD patients and MSA patients. Categorical data were compared with chi square. Probability values less than 0.05 were considered statistically significant.

## 3 RESULTS

### 3.1 Clinical characteristics ‐tremor, bradykinesia, cerebellar ataxic gait

First of all, in terms of clinical characteristics, the clinical data of 30 PD patients, 22 MSA‐P patients, and 28 MSA‐C patients are shown in Table 1. Patient age, gender, and disease duration did not differ significantly among the three groups. However, we found that significant difference between the PD group and the MSA group (MSA‐P and MSA‐C) in disease severity (UPDRS motor score, MSA‐P 26.91 ± 8.60 MSA‐C 24.68 ± 6.06 versus PD 17.77 ± 6.98; *p* < .05, and H‐Y stage, MSA‐P 2.73 ± 0.63 MSA‐C 2.76 ± 0.60 versus PD 1.93 ± 0.72; *p* < .05).

We further identified the clinical symptoms and signs for differentiating these diseases. Tremor or bradykinesia was the initial feature in all PD patients and the majority (86.4%) of MSA‐P patients, but in only one (3.6%) of 28 MSA‐C patients. Though tremor predominated in PD patients, bradykinesia occurred more frequently in MSA‐P (Table 2). In contrast, the majority (53.6%) of MSA‐C patients presented an initial symptom of postural instability, which did not occur in PD and MSA‐C patients (Table 2). At least one of the motor symptoms including tremor at rest, postural tremor, rigidity, bradykinesia, and postural instability developed at latest follow‐up in all the PD, MSA‐P, and MSA‐C patients (Table 3). Compared with the PD group, tremor at rest occurred less frequently, and postural instability was more frequently in MSA‐P and MSA‐C group (*p* < .003). Parkinsonism symptoms such as tremor at rest, rigidity, and bradykinesia were less common in MSA‐C patients than those in PD patients (*p* < .05). There was no significant difference in rigidity, bradykinesia, and postural tremor between PD group and MSA‐P group. In addition, other symptoms were present in MSA group, but not in PD group. Fall and Pisa sign occurred in 2 (9.09%) of 22 MSA‐P patients, and cerebellar ataxic gait, limb ataxia, and dysarthria were present in 25 (89.3%), 24 (85.7%), and 8 (28.6%) of 28 MSA‐C patients, respectively.

**TABLE 2:** Symptoms and signs at onset of the patients

Groups	PD (*n* = 30)	MSA‐P (*n* = 22)	MSA‐C (*n* = 28)
Tremor	28	7*	1*
Bradykinesia	2	12*	0^#^
Postural instability	0	0	15^*^
Dizziness	0	1	4
Urinary incontinence	0	1	3
Unstable holding things	0	0	3
Frequent fall	0	1	0
Speech problem	0	0	2

* *p* < .05 versus PD.

^#^ *p* < .05 versus MSA‐P.

**TABLE 3:** The Parkinson's symptoms in the PD, MSA‐P, and MSA‐C groups

Groups	PD (*n* = 30)	MSA‐P (*n* = 22)	MSA‐C (*n* = 28)
Tremor at rest (%)	28 (93.33)	6 (27.27)*	4 (14.29)*
Postural tremor (%)	19 (63.33)	18 (81.82)	14 (50.00)
Rigidity (%)	28 (93.33)	16 (72.72)	10 (35.71)*
Bradykinesia (%)	21 (70.00)	18 (81.82)	11 (39.29)*
Postural instability (%)	4 (13.33)	11 (50.00)*	28 (100.00)*

* *p* < .003 versus PD.

Compared with PD patients, parkinsonian signs in MSA patients were more frequently symmetric. Only 24% of MSA patients had a good response to levodopa (375–750 mg/day) and the response did not last for more than one year. In addition, autonomic dysfunction was present in 80% of MSA patients, but was in only 13.3% of PD patients (*p* < .05). Dementia occurred in 6% of MSA patients.

### 3.2 MRI findings‐Cerebellum, basal ganglia

**FIGURE 1:**
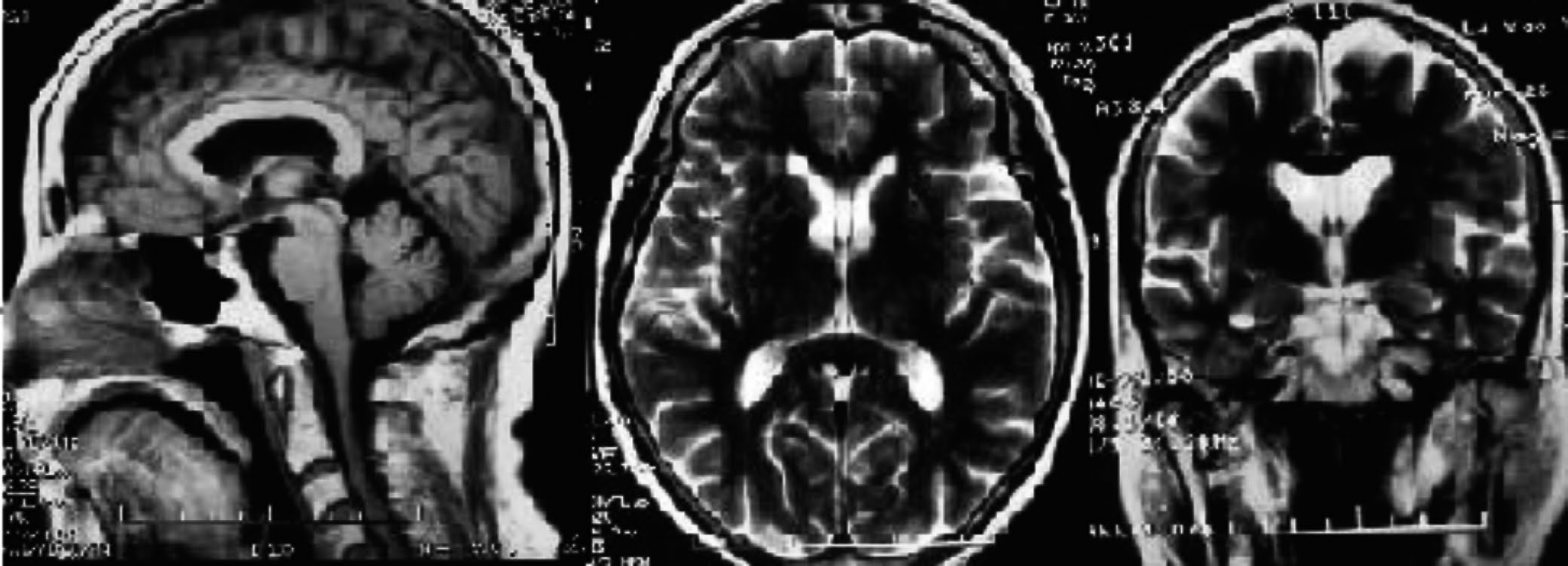
MRI images showing bilateral putamen atrophy and putaminal hyperintensive rim in one MSA‐P patient

**FIGURE 2:**
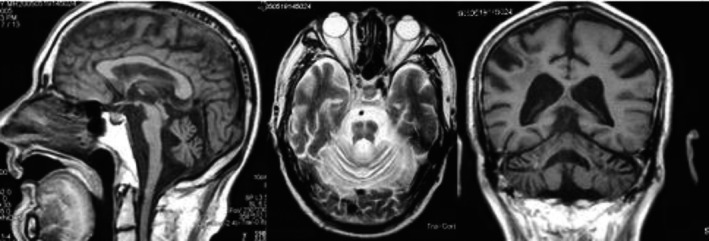
MRI images showing atrophy in the cerebellum and brainstem, “hot cross bun” sign in T_2_WI, enlargement of the fourth ventricle, and mild atrophy in supratentorial structures

Then, we used MRI to do research and found that MRI images showed different signal change among PD, MSA‐P, and MSA‐C groups in infratentorial parameters (cerebellar atrophy, signal increase in the middle cerebellar peduncle, and “hot cross bun” sign), and supratentorial parameters (signal decrease in the basal ganglia, putaminal hyperintensive rim, and cortical atrophy) (Table 4). Putaminal hyperintensive rim was significantly prominent in the MSA‐P group, compared with the PD group and the MSA‐C group (Figure 1a). There was no significant difference in other parameters between PD and MSA‐P groups. Infratentorial parameters were more common in MSA‐C groups than those in PD and MSA‐P groups (Figure 1b). Among the infratentorial parameters, cerebellar atrophy was most frequently found in the MSA‐C patients. However, signal decrease in the basal ganglia and cortical atrophy were not significantly different among these three groups (Figure 2).

**TABLE 4:** MRI signal change in PD, MSA‐P, and MSA‐C groups

Groups	PD (*n* = 30)	MSA‐P (*n* = 22)	MSA‐C (*n* = 28)
Cerebellar atrophy (%)	2 (6.7)	4 (18.2)	17 (60.7)^*^
Signal increase in the middle cerebellar peduncle (%)	0 (0)	1 (4.6)	8 (28.6)^*^
“Hot cross bun” sign (%)	0 (0)	0 (0)	15 (53.6)^*^
Signal decrease in the basal ganglia (%)	8 (26.7)	6 (27.3)	6 (21.4)
Putaminal hyperintensive rim (%)	2 (6.7)	7 (31.8)*	0 (0)^#^
Cortical atrophy (%)	4 (13.3)	4 (18.2)	5 (17.9)

* *p* < .05 versus PD.

^#^ *p* < .05 versus MSA‐P.

### 3.3 ^18^F‐FDG‐PET imaging ‐ Cerebral cortex, basal ganglia, Cerebellum

In order to further explore the differences among these diseases, we also used 18F‐FDG‐PET technology to study. In all patients, 20 PD patients (14 males, age 62.5 ± 11.7 years), 11 MSA‐P patients (5 males, age 67.4 ± 6.1 years), 13 MSA‐C patients (6 males, age 59.8 ± 8.7 years), and 44 normal controls (24 males, age 62.1 ± 9.8 years) underwent ^18^F‐FDG‐PET studies. Patient age and gender were not significantly different among the four groups. The disease duration did not differ significantly among PD, MSA‐P, and MSA‐C groups (*p* < .05; PD 30.70 ± 22.1 months, MSA‐P 34.5 ± 20.1 months, MSA‐C 27.5 ± 12.5 months).

For visual inspection of ^18^F‐FDG‐PET images, we did not identify a significant different pattern of glucose metabolism between PD group and control group. A clear and symmetrical distribution of the radiotracer was found in the brain areas such as cerebrum, cerebellum, caudate nucleus, lenticular nucleus, and thalamus. A reduction in radiotracer uptake in the basal ganglia and in the cerebellum was found in MSA‐P group and MSA‐C group, respectively.

**FIGURE 3:**
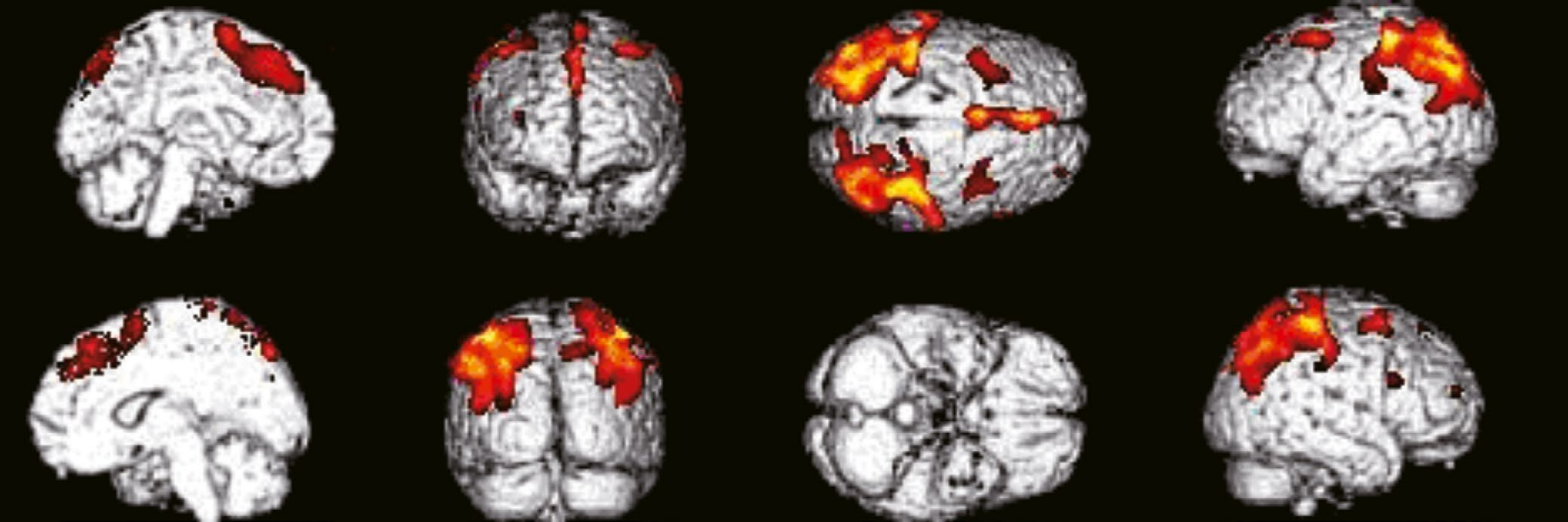
^18^F‐FDG‐PET images in the PD group. Images from SPM analysis showing that in the PD group, glucose metabolism was decreased in bilateral parietal lobe, bilateral frontal lobe, bilateral precuneus, bilateral middle temporal sulcus occipital, and right inferior temporal sulcus and superior occipital sulcus. The hallmark of glucose metabolism is a decreased metabolism in bilateral parietal lobe

We performed SPM analysis of the group differences in ^18^F‐FDG‐PET images among the PD, MSA‐P, and MSA‐C groups. In the PD group, the hallmark of glucose metabolism was a decreased metabolism in parietal areas. 11 (55.0%) of 20 PD patients showed a bilateral reduction in glucose metabolism. Except one patient who showed a reduction in glucose metabolism only in bilateral parietal area, hypometabolic areas in these patients were also observed in parieto‐occipital, frontal, occipital, and temporal cortical areas in 3, 3, 2, and 2 PD patients, respectively. 3 patients showed parieto‐occipital reduction in glucose metabolism. In addition, 3 PD patients showed different metabolic patterns. One patient had a unilateral reduction in glucose metabolism in frontal lobe, putamen, thalamus, cerebellum, and pons. 2 patients exhibited a decreased metabolism in right putamen and bilateral cerebellum. The glucose metabolism was not significantly different in 3 other PD patients compared with controls (Figure 3).

**FIGURE 4:**
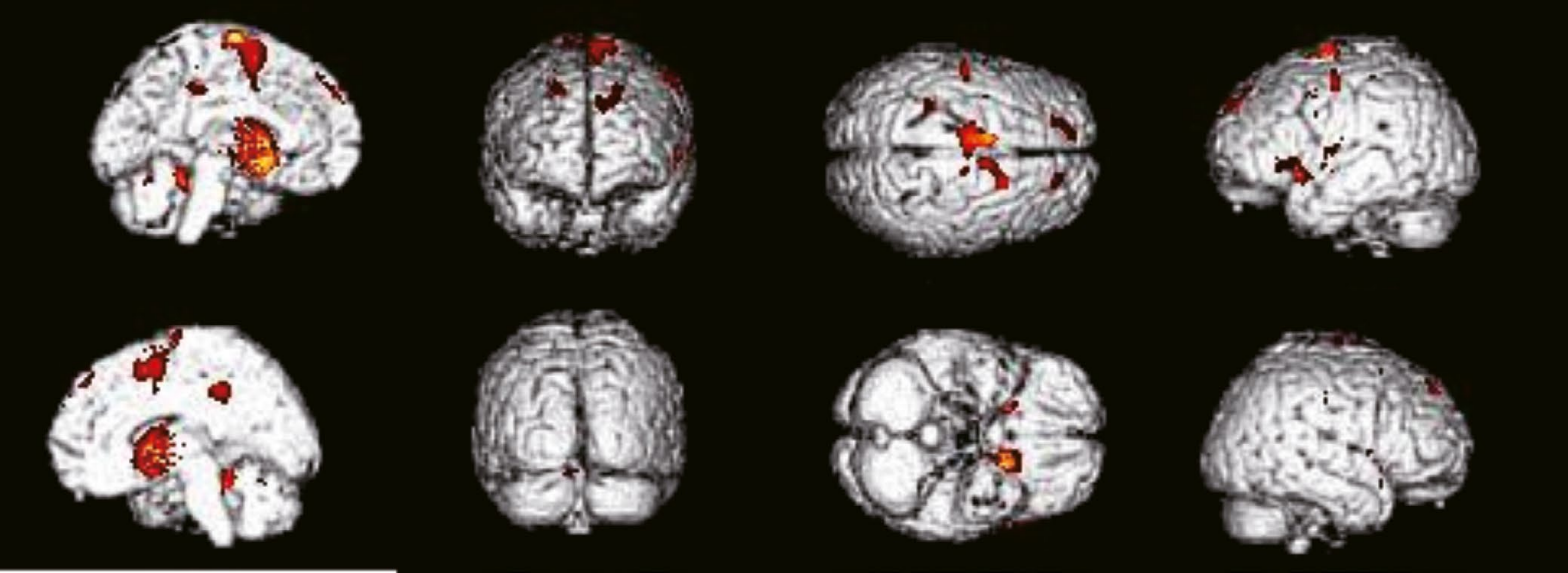
^18^F‐FDG‐PET images in the MSA‐P group. Images from SPM analysis showing that in the MSA‐P group, glucose metabolism was decreased in bilateral caudate nucleus, bilateral cingulated cortex, left putamen, left inferior parietal lobule and posterior central sulcus, and right superior temporal sulcus and anterior central sulcus. The distinguishing feature of the MSA‐P group is the presence of a global hypometabolism in bilateral basal ganglia

A distinguishing feature of the MSA‐P group was the presence of a global hypometabolism in bilateral putamen and caudate. This finding was found in 8 of 11 patients, and 3 of them also had a mild and local reduction in glucose metabolism in bilateral cerebellum. In addition, hypometabolic areas were also observed in bilateral frontal, parietal, occipital, and insular cortical areas (Figure 4).

**FIGURE 5:**
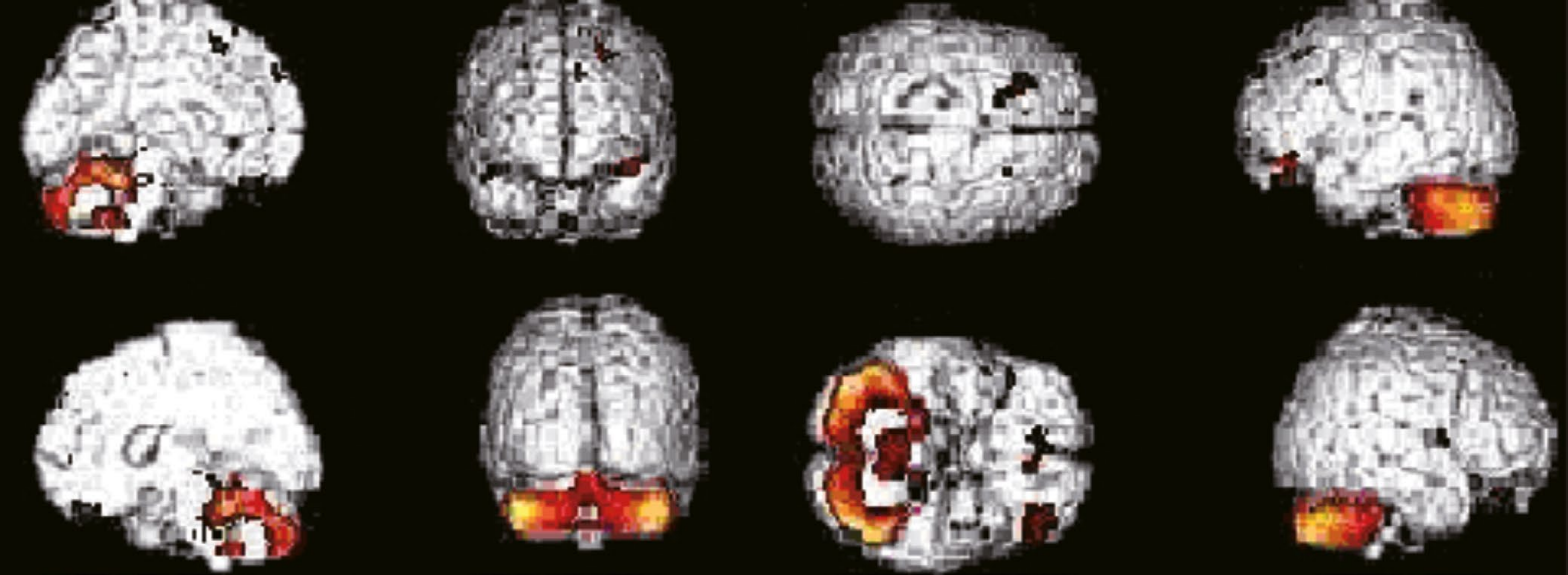
^18^F‐FDG‐PET images in the MSA‐C group. Images from SPM analysis showing that in the MSA‐C group, glucose metabolism was decreased in bilateral cerebellum and medulla oblongata, and bilateral parental and frontal lobe. The distinguishing feature of the MSA‐C group is the presence of a glucose hypometabolism in bilateral cerebellum

The glucose metabolism in the MSA‐C group is characterized by a marked bilateral reduction in cerebellum. All 13 patients had this distinct characteristics, and 8 (61.5%) of them also showed a hypometabolism in the middle cerebellar peduncle. In addition, hypometabolic areas were also observed in pons and oblongata, frontal area, parietal, and occipital area in 1, 5, and 2 of MSA‐C patients, respectively (Figure 5).

## 4 DISCUSSION

In this study, we seek to study the PD, MSA‐P, and MSA‐C, and to find possible discriminating patterns of clinical symptoms, MRI findings, and ^18^F‐FDG‐PET imaging. We identify several main findings that reflected the difference among PD, MSA‐P, and MSA‐C. First, clinical data show that the predominant motor symptom was greatly different in that tremor at rest, bradykinesia, and postural instability predominates in PD, MSA‐P, and MSA‐C patients, respectively. Second, MRI findings indicate that putaminal atrophy and hyperintensive rim (T_2_WI) are characteristic features in MSA‐P, and cerebellar atrophy, “hot cross bun” sign, and signal increase in the middle cerebellar peduncle are more prominent in MSA‐C. Third, the ^18^F‐FDG‐PET images demonstrate that a reduction in glucose metabolism occurs in parietal area for PD, in bilateral putamen for MSA‐P, and in bilateral cerebellum for MSA‐C. This study identifies the characteristic features of clinic symptoms, MRI abnormality, and glucose metabolism on ^18^F‐FDG‐PET, which are indispensable for differential diagnosis among PD, MSA‐P, and MSA‐C.

The clinical presentation, MRI findings, and PET images in our study consistently reflect the pathophysiology of these diseases. It is known that the pathology of MSA‐P predominates in the basal ganglia, while the pathology of MSA‐C predominates in the cerebellum (Ozawa et al., 2004). Bradykinesia is a hallmark of basal ganglia disorders and appears to correlate with the degree of dopamine deficiency (Berardelli, Rothwell, Thompson, & Hallett, 2001; Vingerhoets, Schulzer, Calne, & Snow, 1997). It has been reported that the decreased uptake of F‐fluorodopa is proportional to the degree of bradykinesia (Lozza, Marie, & Baron, 2002; Niccolini & Politis, 2016). Our finding that putaminal hyperintensive rim in MRI and a reduction in glucose metabolism in ^18^F‐FDG‐PET agree with bradykinesia as the dominant clinical feature in MSA‐P group. In contrast, cerebellar signs such as ataxic gait, limb ataxia, and postural instability are more frequently present in MSA‐C patients. This clinical feature is supported by the characteristic features on MRI and ^18^F‐FDG‐PET images showing predominant cerebellar lesions. In addition, at the similar disease duration, MSA‐P and MSA‐C patients exhibit more severity in motor dysfunction with higher UPDRS and H‐Y scores than PD patients, supporting the fact that MSA develops more rapidly than PD (Laurens et al., 2017).

Putaminal hyperintensive rim in the MRI (T_2_WI) occurs most frequently in MSA‐P patients compared with PD and MSA‐C patients. However, hyperintensive rim occurs in only 31.8% of MSA‐P patients and is also present in 2 (6.7%) of PD patients. The low sensitivity disfavors hyperintensive rim as a useful parameter for distinguishing MSA‐P from PD as previously reported (Hughes et al., 2002). The specificity of hyperintensive rim in this study is different from a previous report, which demonstrated that hyperintensive rim showed a highest specificity (90%) in MSA‐P patients (Hughes et al., 2002). This difference may result from different patients with variant clinical features and disease stage as well as different imaging conditions between the two studies. In addition, hyperintensive rim is not present in our MSA‐C patients with average disease duration of about 3 years. It is reported that hyperintensive rim occurs early in MSA‐P patient (less than 3 years) and late in MSA‐C patient (more than 4 years) (Pradhan & Tandon, 2017). Therefore, the presence of hyperintensive rim on MRI may be a good sign to exclude the diagnosis of MSA‐C, especially at the early stage of the disease.

Infratentorial parameters in MRI such as cerebellar atrophy, signal increase in middle cerebellar peduncle, and “hot cross bun” sign show a higher specificity in MSA‐C patients, and are useful for differentiation of MSA‐C from PD and MSA‐P patients. “Hot cross bun” sign exhibits the highest specificity in MSA‐C patients and is not found in MSA‐P patients. This result agrees with previous study that “hot cross bun” sign was observed later than putaminal hyperintensive rim in MSA‐P (Pradhan & Tandon, 2017). However, signal increase in middle cerebellar peduncle occurs only in 28.6% of MSA‐C patients, suggesting that it is not a sensitive parameter for early diagnosis of MSA‐C.

In order to further explore the differences between these diseases, we also used ^18^F‐FDG‐PET for research which detects the change of regional glucose metabolism has been used for differential diagnose of PD from MSA (Grimaldi et al., 2019; Kwon et al., 2007; Meles, Teune, de Jong, Dierckx, & Leenders, 2017; Meyer et al., 2017; Ping, Benshu, & Shuo, 2012; Tomše et al., 2017), since glucose metabolism at resting state is an effective marker for density and activity of synapse, which can reflect the neurodegenerative states of parkinsonian diseases. The glucose metabolism is not decreased in the basal ganglia in the majority of our PD patients with average disease duration of <3 years, suggesting that the basal ganglia are not seriously affected at the early stage of PD. However, glucose hypometabolism was found mainly in the parietal areas in our PD patients with no sign of dementia. This finding agrees with a previous report that the glucose metabolism was decreased in the parietal and parieto‐occipital regions in nondemented Parkinson's patients (Albrecht, Ballarini, Neumann, & Schroeter, 2019). It may be related to the regulation of Nigra striatum dopaminergic pathway in substantia nigra striatum of early Parkinson's disease. In addition, only one patient in MSA‐P and MSA‐C patients exhibits a hypometablosm in parietal area, suggesting that it is a good parameter for differentiation of PD from MSA. However, since 3 PD patients did not exhibit any difference in glucose metabolism from controls, the absence of glucose hypometabolism cannot exclude the diagnosis of PD. However, clinical diagnosis of PD is confirmed correctly by 76% of postmortem cases (Rajput et al., 1991). At present, the diagnosis of early PD is mainly based on clinical symptoms, and there is no specific manifestation of head MRI in the early stage. In this study, 11/20 patients with clinically diagnosed Parkinson's disease had bilateral parietal hypometabolism, and another 3/20 patients had hypometabolism in the parieto‐occipital syndesmosis area, which was not high, possibly due to the following reasons: (a) In this study, the duration of Parkinson's disease was 42.93 ± 22.11 months, which was relatively short. Perhaps, the typical manifestations would gradually appear in patients with Parkinson's disease over time; (b) 3/20 patients had hypometabolism in the occipitoparietal junction area, and the clinical manifestations and FDG‐PET manifestations should be dynamically observed to further determine the diagnosis of PD. (c) One patient had a unilateral reduction in glucose metabolism in frontal lobe, putamen, thalamus, cerebellum, and pons. 2 patients exhibited a decreased metabolism in right putamen and bilateral cerebellum. The presence or absence of Parkinson's superimposition in these three patients should be further diagnosed by dynamic observation of PDG‐PET changes. (d) The glucose metabolism was not significantly different in 3 other PD patients compared with controls. Whether PDG‐PET changes of PD or MSA will occur gradually with the progression of the disease should be followed up. These findings suggest that the diagnosis of Parkinson's disease should be a dynamic process, and perhaps early diagnosis of Parkinson's disease patients will continue to revise the diagnosis as the disease progresses.

Our study also shows that glucose hypometabolism mainly occurs in bilateral putamen for MSA‐P patients and in cerebellum for MSA‐C patients. This characteristic of PET image is consistent with neuropathologic feature of MSA‐P (predominated in basal ganglia) and MSA‐C (predominated in cerebellum) (Ozawa et al., 2004). The reduction in glucose metabolism in bilateral putamen on PET occurs in 72.7% of MSA‐P patients and only in 2 (10.0%) of 20 PD patients, suggesting that it has a good parameter for differential diagnosis of MSA‐P and PD. In addition, this finding also suggests that hypometabolism in putamen on PET (72.7%) is more sensitive than the hyperintensive rim in MRI (31.8%) for diagnosis of MSA‐P. Furthermore, the hypometabolism in cerebellum shows the highest specificity in diagnosis of MSA‐C, since this feature is present in all MSA‐C patients, but not in PD patients. Since 3 MSA‐P patients also show a reduction in glucose metabolism in cerebellum, suggesting that cerebellar injury may occur very early in certain patients. Some studies suggest that glial cytoplasmic inclusions (GCIs) are present in the cortex of MSA patients, especially in the early frontal and parietal lobes (Papp & Lantos, 1994). Our study found that besides the decrease of subtentorial structural metabolism, there was a decrease of cerebral cortical glucose metabolism in the early stage of MSA. It was also observed that cognitive dysfunction in MSA patients was mainly manifested in poor vocabulary memory and executive function (Burk, Daum, & Rub, 2006), which was the manifestation of prefrontal lobe damage, consistent with the manifestation of frontal lobe involvement in PET. In this study, the average duration of the disease was less than 3 years, and further observation of clinical symptoms and the development of PET should be conducted to understand the cortical damage.

### 4.1 Limitation

There are some limitations in this study. For example, the sample size of this study is not very large. Also, the evaluation indicators need to be further expanded to better understand the differential diagnosis among PD, MSA‐P, and MSA‐C.

## 5 CONCLUSION

In summary, we identify the characteristic features of PD, MSA‐P, and MSA‐C in clinical feature, MRI findings, and PET images. The differences among PD, MSA‐P, and MSA‐C are consistent with the pathological differences of these diseases, and thus appears to be useful for differential diagnosis among them. In the early stage of the disease, the clinical symptoms and signs are atypical, and the positive rate of MRI imaging is not high, which often brings difficulties to the diagnosis. ^18^F‐FDG‐PET imaging can find more typical disease signs, which is a better differential diagnosis method.

## CONFLICTS OF INTEREST

All authors have contributed significantly to the manuscript and declare that the work is original and has not been submitted or published elsewhere. None of the authors have any financial disclosure or conflict of interest.

## AUTHORS' CONTRIBUTIONS

PZ and BZ conceptualized and designed the study, drafted the initial manuscript, and reviewed and revised the manuscript. BZ, SG, and XL designed the data collection instruments, collected data, carried out the initial analyses, and reviewed and revised the manuscript. PZ coordinated and supervised data collection, and critically reviewed the manuscript for important intellectual content. All authors approved the final manuscript as submitted and agree to be accountable for all aspects of the work.

### Peer Review

The peer review history for this article is available at https://publons.com/publon/10.1002/brb3.1827.

## Data Availability

The datasets used and/or analyzed during the current study available from the corresponding author on reasonable request.
